# Redox Regulation of Nrf2 in Cisplatin-Induced Kidney Injury

**DOI:** 10.3390/antiox12091728

**Published:** 2023-09-06

**Authors:** Kranti A. Mapuskar, Casey F. Pulliam, Diana Zepeda-Orozco, Benjamin R. Griffin, Muhammad Furqan, Douglas R. Spitz, Bryan G. Allen

**Affiliations:** 1 Free Radical and Radiation Biology Program, Department of Radiation Oncology, University of Iowa Hospitals & Clinics, Iowa City, IA 52242, USA; 2 Pediatric Nephrology and Hypertension at Nationwide Children’s Hospital, Columbus, OH 43205, USA; 3 Kidney and Urinary Tract Center, Abigail Wexner Research Institute, Nationwide Children’s Hospital, Columbus, OH 43205, USA; 4 Department of Pediatrics, The Ohio State University, Columbus, OH 43210, USA; 5 Division of Nephrology, The University of Iowa Hospitals and Clinics, Iowa City, IA 52242, USA; 6 Department of Internal Medicine, The University of Iowa Hospitals and Clinics, Iowa City, IA 52242, USA

**Keywords:** oxidative stress, reactive oxygen species, Nrf2, chemotherapy, kidney injury, metabolism, mitochondria, superoxide

## Abstract

Cisplatin, a potent chemotherapeutic agent, is marred by severe nephrotoxicity that is governed by mechanisms involving oxidative stress, inflammation, and apoptosis pathways. The transcription factor Nrf2, pivotal in cellular defense against oxidative stress and inflammation, is the master regulator of the antioxidant response, upregulating antioxidants and cytoprotective genes under oxidative stress. This review discusses the mechanisms underlying chemotherapy-induced kidney injury, focusing on the role of Nrf2 in cancer therapy and its redox regulation in cisplatin-induced kidney injury. We also explore Nrf2's signaling pathways, post-translational modifications, and its involvement in autophagy, as well as examine redox-based strategies for modulating Nrf2 in cisplatin-induced kidney injury while considering the limitations and potential off-target effects of Nrf2 modulation. Understanding the redox regulation of Nrf2 in cisplatin-induced kidney injury holds significant promise for developing novel therapeutic interventions. This knowledge could provide valuable insights into potential strategies for mitigating the nephrotoxicity associated with cisplatin, ultimately enhancing the safety and efficacy of cancer treatment.

## 1. Introduction

Chemotherapy remains a central pillar in cancer treatment, yet its effects can extend beyond cancer cells, impacting normal tissues such as the brain [1,2], liver [3,4,5], pancreas [6,7], and kidneys [8]. Platinum-based chemotherapies, particularly cisplatin, can initiate kidney injury, culminating in acute and chronic changes. These changes can hinder drug clearance, prompt treatment setbacks, and impose restrictions that contribute to acute kidney injury (AKI), chronic kidney disease (CKD), and an elevated risk of developing end-stage renal disease (ESRD) [9,10,11,12]. Consequently, the ramifications of cisplatin-induced kidney injury have substantial influence over both overall prognosis and patient wellbeing. 

Reactive Oxygen Species (ROS) are byproducts of normal cellular metabolism that play a crucial role in several physiological processes [13,14,15]. Increased steady-state levels of ROS lead to oxidative stress that has been implicated in chemotherapy-induced kidney injury [16]. In the context of kidney injury, increased levels of ROS can be a result of impairment in the proteins of the mitochondrial electron transport chain [16], NADPH oxidases (NOXs) [17], nitric oxide synthase [18,19,20], and inflammatory pathways [21,22]. Furthermore, increased steady-state levels of ROS can oxidize and thus damage lipids, DNA, and proteins promoting inflammation and activating profibrotic pathways that can cause the accumulation of extracellular matrix proteins (ECM) leading to renal fibrosis. In contrast, increased oxidative stress can activate several redox signaling response cascades including nuclear factor erythroid 2-related factor (Nrf2) mitogen-activated protein kinases (MAPK) and nuclear factor-kappa-B (NF-κB) that in turn regulate gene expression, inflammation, stress-induced immune response, and repair pathways [23,24]. 

Nuclear factor erythroid 2-related factor (Nrf2; encoded by NFE2L2) was discovered in 1994 as a member of the cap “n” collar (CNC) subfamily of basic region leucine zipper (bZip) transcription factors [25]. Subsequent work has established a role for Nrf2 in cellular antioxidant defenses and maintenance of redox homeostasis by regulating the induction of a wide array of antioxidants and drug-metabolizing enzymes including superoxide dismutase (SOD), catalase (CAT), glutathione peroxidase (GPx), hemoxygenase (HO-1), NAD(P)H Quinone Dehydrogenase 1 (NQO-1), and glutathione S-transferase (GST) [23,25,26]. Regulation of the Nrf2 pathway by redox signaling ensures necessary cellular alterations by a network of enzymes that help combat the increased oxidative stress via activation of repair mechanisms to counteract the chemotherapy-induced oxidative damage. Understanding the role of Nrf2 and its regulatory targets can have pivotal implications in several pathologies that are associated with oxidative stress including kidney injury and cancers. This review will discuss several aspects of cisplatin-induced kidney injury, focusing on the Nrf2 signaling pathway and its redox regulation. We will also discuss the downstream signaling components of Nrf2 that play a role in antioxidant response to chemotherapy-induced kidney injury as well as highlight the preclinical and therapeutic implications of enhancing the activity of Nrf2 and its effects on chemotherapy-induced kidney injury. 

## 2. Chemotherapy-Induced Kidney Injury

The development of nephrotoxicity is a common side effect of several chemotherapeutic agents causing adverse structural as well as functional changes in the kidney contributing to the pathology of kidney disease. The drugs most associated with chemotherapy-induced renal implications include cisplatin [27,28,29], ifosfamide [30,31,32,33], methotrexate [34,35], cyclophosphamide [11,36], Bevacizumab [37], streptozotocin [38], and gemcitabine [39].

One of the most used chemotherapy drugs is cisplatin (cis-diamminedichloroplatinum II), a platinum-containing antineoplastic drug extensively used to treat a variety of cancers and notorious for its toxic effects on the kidney. Being one of the most potent and effective chemotherapies, cisplatin is used to treat testicular, cervical, breast, small cell lung, and bladder cancers [40,41,42,43]. It is broadly accepted that cisplatin exerts its damaging effects by causing intra or interstrand crosslinks with purine bases on DNA which impairs DNA repair pathways causing cell cycle arrest and ultimately cell death [44,45]. Regardless of the effectiveness of cisplatin in sensitizing a plethora of cancers, its usage is rationed due to its adverse nature causing ototoxicity [46,47,48], nephrotoxicity [45,49], and neurotoxicity [50,51]. Cisplatin enters the renal tubular cells through its uptake transporters (OCT2) and (CTR1) and forms reactive metabolites that cause DNA damage, and oxidative stress, and disrupt cellular function [52,53]. Cisplatin also affects the resorption of electrolytes by tubular cells leading to electrolyte imbalances, an increase in serum creatinine, fluid retention, increased intratubular pressure, and reduced urine output, all of which lead to renal functional decline and are classic characteristics of Acute Kidney Injury (AKI) [52,54]. Higher cumulative doses of cisplatin and recurrent episodes of AKI can increase the risk of developing CKD [55]. Risk factors for cisplatin include pre-existing renal conditions, old age, previous dosing with cisplatin, and genetic factors that affect the metabolism of the drug in the kidney and its elimination [56]. 

Methotrexate (MTX) is an antifolate therapy, most commonly used in the treatment of rheumatoid arthritis (RA) [57], psoriasis [58], inflammatory bowel disease, and a variety of cancers, including lung, breast, and leukemia. The primary mechanism of action involves the inhibition of dihydrofolate reductase (DHFR) which is involved in purine and pyrimidine synthesis [57], the building blocks for DNA and RNA. The usage of methotrexate is limited due to nephrotoxicity with high doses of MTX (>500 mg/m^2^) inducing AKI through a mechanism involving oxidative stress and inflammation leading to apoptosis [59,60,61].

Cyclophosphamide, an antineoplastic agent with a cytotoxic and immunosuppressive profile is often used to treat autoimmune disease as well as systemic lupus [62]. It is also used to treat lymphomas, leukemia, and certain type of solid tumors. Cyclophosphamide is converted to its active metabolites by cytochrome p450s in the liver and primarily causes nephrotoxicity due to its metabolite, phosphoramide mustard, that produces the inter- and intrastrand DNA crosslinks [63]. Although considered an effective anticancer agent, cyclophosphamide can have detrimental effects on the kidneys including hemorrhagic cystitis, tubular toxicity, and glomerular toxicity [11]. 

Ifosamide (IFO), an analog of cyclophosphamide, is also an alkylating agent used to treat soft tissue sarcomas, testicular cancers, and lymphomas, and can be nephrotoxic at high doses [64,65]. IFO requires metabolic activation by cytochrome p450s to be converted to a 4-hydroxy form which in turn spontaneously releases the active form, isophosphoramide mustard [66]. Other parallel pathways for IFO metabolism include its inactive metabolites (2-dechloroethylifosfamide and 3-dechloroethylifosfamide) [66]. IFO can cause renal tubular dysfunction causing hypokalemia, hypophosphatemia, and several other electrolyte imbalances [67]. Furthermore, it can manifest as urinary excretion of glucose, bicarbonate, and amino acids [67]. Additionally, it can promote tubular interstitial injury that can present as AKI or in some cases progressive CKD [68]. IFO and its metabolites can also cause inflammation of the bladder leading to hemorrhagic cystitis which can result in bladder pain, bloody urine, and urinary frequency [69]. 

Doxorubicin, an anthracycline drug first extracted from Streptomyces peucetius var. caesius in the 1970s is used in breast cancer, lymphomas and lung cancer [70]. While treatment with doxorubicin although primarily affects the cardiovascular system, there are renal implications including glomerular and tubular damage, nephrotoxicity, and AKI [71]. Although the primary mechanism of doxorubicin-induced nephrotoxicity is not fully understood, several studies have elucidated a role for oxidative stress, inflammation, and direct renal toxicity with damage to the glomerular structure resulting in proteinuria, impaired renal function, and AKI [70,71]. It has been noted that patients receiving higher cumulative doses of doxorubicin or those with pre-existing renal conditions may be at a higher risk of developing renal injury [72].

Kidney toxicity caused by anticancer, chemotherapeutic agents is often associated with higher doses or prolonged drug exposure. Additional factors that affect the presentation and degree of kidney injury include age, sex, and comorbidities.

Chemotherapy-induced kidney injury can occur through direct mechanisms whereby drugs directly damage renal cells in the tubules and glomeruli (e.g., Cisplatin, methotrexate, cyclophosphamide, and ifosfamide). Several chemotherapy drugs can also induce oxidative stress and trigger an inflammatory response resulting in kidney injury. Impaired blood flow and oxygen delivery to renal tissue can lead to renal tubulointerstitial hypoxia and vascular damage which can disrupt the normal functioning of the kidney, thus contributing to renal impairment [73,74]. Immune checkpoint inhibitors (ICIs) used in the management of many advanced cancers can result in acute interstitial nephritis [75]. In addition, chemotherapy-induced renal dysfunction has been linked to mitochondrial structural and functional alterations that can present as dysregulation of the mitochondrial electron transport chain complexes, altered mitochondria membrane potential as well as reduced ATP levels and increased generation of reactive oxygen species (ROS) [16,53,76]. Increased levels of ROS generated from oxidative phosphorylation and/or NAPDPH oxidases can impair renal homeostasis and function, leading to inflammation, tissue damage, and fibrosis [77]. Endogenous cellular antioxidant systems effectively alleviate these ROS increases, thus maintaining the redox status (balance between pro-oxidants and antioxidants) of cellular systems. 

## 3. Role of Nrf2 in Antineoplastic Therapy

Nrf2, known for its role in counteracting oxidative stress, also significantly contributes to managing overall toxicity and mitigating adverse reactions arising from the formation of antineoplastic DNA adducts. This is achieved by initiating the expression of detoxification genes, notably UDP-glucuronosyltransferases (UGTs), glutathione S-transferases (GSTs), and quinone reductases (NQOs) [78]. These genes collectively facilitate the removal of toxic compounds and xenobiotics, concurrently supporting broader cellular metabolism. Furthermore, the increased expression of genes associated with DNA damage repair pathways effectively prevents the accumulation of DNA adducts, thus reducing the risk of undesirable effects linked to DNA damage resulting from antineoplastic agents. A 2015 study by Jayakumar et al. revealed Nrf2’s facilitation of radiation-induced DNA damage repair through a homologous recombination repair pathway in cancer cells [79]. Remarkably, this repair mechanism operates independently of ROS. The inhibition of Nrf2 substantially impeded DNA repair, leading to diminished RAD51 mRNA levels—an essential participant in homologous recombination and DNA repair—resulting in persistent DNA damage even in the presence of N-acetyl cysteine (NAC), a free radical scavenger [79]. This underscores the broader influence of Nrf2 beyond its antioxidant functions. Additionally, other investigations have shown Nrf2’s capacity to enhance DNA repair via phosphorylation at serine-40 by the DNA damage response kinase, Ataxia Telangiectasia Mutated (ATM), in response to DNA damage [80]. 

With regards to antineoplastic strategies, including chemotherapeutic agents like cisplatin, the induction of DNA adduct formation is one mechanism to target rapidly dividing cancer cells. However, the lack of selectivity inherent to this approach leads to unintended damage to normal cells resulting in adverse reactions. The activation of Nrf2, leading to the augmented expression of genes associated with DNA repair and detoxification pathways, effectively contributes to the proficient removal of DNA adducts from both cancerous and normal cells. Consequently, this reduction in DNA damage-associated adverse effects stemming from antineoplastic treatments is substantial. Nonetheless, a cautious approach is imperative when considering Nrf2 modulation and activation. While its protective role is well established, prolonged and excessive Nrf2 activation may inadvertently bolster cancer cell survival and promote chemoresistance. Hence, any manipulation of Nrf2 and its activation necessitates careful consideration within the context of therapeutic strategies.

## 4. Signaling and Post-Translational Modifications of Nrf2

Increased oxidative stress is one of the most common causes of cisplatin-induced kidney injury, often presenting as altered oxidative phosphorylation, altered mitochondrial metabolism, upregulation of NADPH oxidases, and ROS generation [16]. A simultaneous decrease in the levels of antioxidants further enhances ROS levels contributing to an altered redox state. Under such conditions, cells activate transcription factors that can induce antioxidant enzymes that can combat the increased levels of ROS thus protecting against oxidative stress [81,82]. Nrf2 is a redox-sensitive CNC transcription factor that contains a bZip domain known to modulate cellular oxidative stress [83,84]. Kelch-like ECH-associated protein 1 (Keap1) previously identified in 1999 as a repressor of Nrf2 was subsequently classified as a E3 ubiquitin ligase that targets Nrf2 for degradation [85,86]. Under homeostatic conditions, Nrf2 is sequestered in the cytoplasm by Keap1, regulating the activity of Nrf2 by targeting it for ubiquitination and proteasome-dependent degradation [86,87]. Keap1 thus serves as a sensor for redox changes and acts as a negative regulator of Nrf2 [87]. Under high oxidative stress levels, modifications to specific sensor cysteine residues on Keap1 lead to disruption of the interaction between Nrf2 and Keap1, allowing Nrf2 to translocate into the nucleus where it can promote the transcription of several antioxidant enzymes [87] including thioredoxin reductase 1 (TXNRD1), glutathione S-transferase A1 (GST), NAD(P)H quinone oxidoreductase 1 (NQO1), heme oxygenase 1 (HO-1), glutamate–cysteine ligase modifier subunit (GCLM), and a catalytic subunit (GCLC) [88,89,90]. Once in the nucleus, Nrf2 binds to antioxidant response elements (AREs) present in the promoter regions of target genes, initiating their transcription and synthesizing various antioxidant and detoxification enzymes, thereby enhancing cellular defense against oxidative stress.

**Figure 1:**
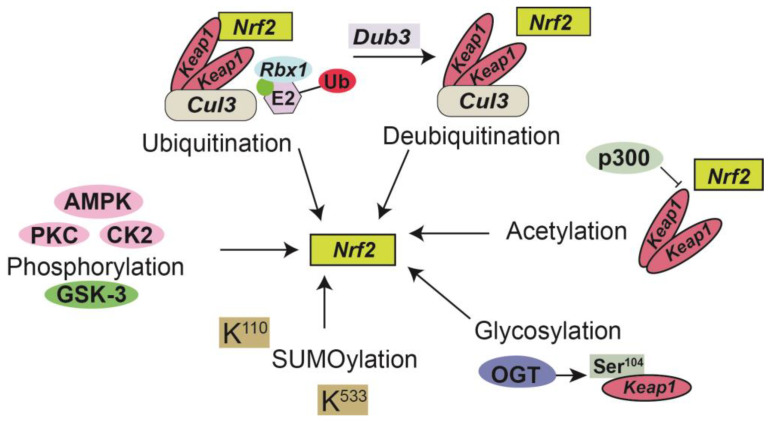
Post-translational modifications of Nrf2. Phosphorylation by kinases in the post-translational regulation of NRF2. Protein kinase C (PKC), Casein kinase 2 (CK2), and AMP-activated kinase (AMPK) positively regulate Nrf2 (pink); glycogen synthase kinase 3 (GSK-3) negatively regulates Nrf2 activity (green) through phosphorylation at different sites [81]. Conjugation of the Cul3-Keap1-E3 ligase with Nedd8 (green circle) leads a Keap1 homodimer to recruit Nrf2 to the Cul3-Keap1-E3 ligase complex and target it for ubiquitination [90]. Deubiquitinase3 (DUB3) promotes the stability and transcriptional activation of Nrf2 [95]. The acetyltransferase p300 is a part of the Nrf2 transcriptional complex that promotes its nuclear accumulation by interfering with the formation of the Nrf2-Keap1 complex [91]. KEAP1 is glycosylated at serine 104 by O-GlcNAc transferase (OGT) [93] subsequently activating Nrf2. Small ubiquitin-like modifier (SUMO) proteins alter Nrf2 at K^110^ and K^533,^ promoting its nuclear localization [92].

Post-translational modifications (PTMs) are highly dynamic, reversible, and covalent expansions that are a series of specific enzyme-catalyzed modifications to a protein’s side chain or backbone [91]. PTMs have been implicated as biomarkers and/or mediators of renal injury including CKD [92] and are involved in multiple aspects of Nrf2 regulation including phosphorylation [84], ubiquitination [93], acetylation [94], SUMOylation [95], and glycosylation [84,92,96] (Figure 1). Phosphorylation can lead to modifications of NRF2, depending on the phosphorylation site for various protein kinases including AMPK, PKC, GSK-3, CDK5, and MAPK thus affecting nuclear translocation, proteasomal degradation, ARE binding, and nuclear export of Nrf2 [84]. For example, PKC-induced phosphorylation has been shown to prevent Keap1-mediated degradation of Nrf2. In contrast, GSK-3-induced phosphorylation destabilizes Nrf2. Furthermore, AMPK phosphorylates Nrf2 and prevents its nuclear export [84]. Nrf2 can also undergo ubiquitination by Keap1 leading to its proteasomal degradation by a Cul3 (cullin3)-mediated ubiquitination complex [97]. In contrast, deubiquitinase3 (DUB3) promotes the stability and transcriptional activation of Nrf2 by decreasing the K48-linked ubiquitination of Nrf2 [98]. Additionally, KEAP1 is glycosylated at serine 104 by O-GlcNAc transferase (OGT) [93], subsequently activating Nrf2, thus establishing a link between Nrf2 signaling and nutrient sensing [99]. Studies performed by Chen et al. identify KEAP1 as a substrate for O-GlcNAc transferase (OGT), which adds the post-translational modification (PTM) O-GlcNAcylation at S104 [99]. SUMO-2 mediated SUMOylation of specific lysine residues (K110 and K533) on Nrf2, in part enhances stabilization and can regulate its nuclear localization [95]. Recent reports have also suggested that the acetylation of Nrf2 by p300/CBP increases promoter-specific DNA binding of Nrf2 and directly acetylated Nrf2 in response to arsenite-induced stress [100].

## 5. Redox Regulation of Nrf2 in Cisplatin-Induced Kidney Injury 

While effective, commonly used chemotherapeutic agents like cisplatin can impose detrimental side effects on normal tissues, including the kidneys. One of the crucial mechanisms underlying cisplatin-induced kidney toxicities is the impairment of redox homeostasis. Due to the comprehensive nature of the several proteins encoded by Nrf2 (antioxidants, detoxifying enzymes, and anti-inflammatory proteins) and their combined effects on redox metabolism, the Nrf2 pathway and its downstream targets are deemed essential for protection against several cisplatin-induced redox pathologies. 

**Figure 2:**
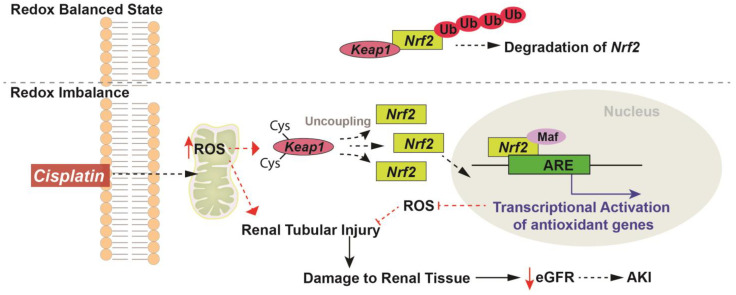
Redox regulation of Nrf2 in cisplatin induced injury. Under a redox balanced state, Nrf2 is bound to Keap1 and targeted for degradation by proteosome-mediated ubiquitination. Chemotherapy agents like cisplatin once inside the cell can lead to mitochondrial dysfunction [16] and increase ROS levels that can stimulate apoptotic mechanisms leading to renal tubular damage, decreased estimated glomerular filtration rate (eGFR), and manifestation of renal pathology that can lead to the development of acute kidney injury (AKI). Increased ROS levels oxidize the cysteine residues on Keap1 and lead to its uncoupling from Nrf2. Once uncoupled, Nrf2 translocates to the nucleus and binds the Maf (musculoaponeurotic fibrosarcoma) protein (has a leucine zipper (Zip) domain required for heterodimer complex formation) to form the Nrf2–Maf complex which subsequently binds to the antioxidant response element (AREs) that promotes the transcriptional activation of several cytoprotective and antioxidant genes. Increased expression and activity of antioxidants in the cells can alleviate the increases in ROS levels subsequently mitigating the overall renal injury.

Activation of Nrf2 and its downstream effectors has been pivotal in alleviating oxidative stress associated damage in cells and tissues, making the Nrf2 signaling pathway an attractive therapeutic target [101,102]. Once activated, Nrf2 is translocated to the nucleus where it forms a heterodimer with the leucine zipper domain containing Maf (musculoaponeurotic fibrosarcoma) protein at the bZip DNA binding site which is bound by the Maf oncogene to recognize and subsequently bind the AREs leading to the transcriptional activation of several cytoprotective genes including antioxidants, phase II detoxification enzymes and other factors that aid in restoring redox homeostasis as well as reduce inflammation and sensitivity (Figure 2) [86,87]. Several studies have shown the upregulation of antioxidant genes controlled by Nrf2 including HO-1 [103], NQO1 [104], GPx [105,106], and SOD [16,53] in cisplatin-induced renal injury [107]. Bolisetty et al. demonstrated in proximal tubule-specific Cre transgenic mice that the deletion of HO-1, specifically in the proximal tubules, augments structural and functional damage during cisplatin nephrotoxicity and that selective overexpression of HO-1 in proximal tubules is protective [103]. Furthermore, HO-1 KO mice demonstrated an increased level of cleaved caspase-3 expression, and p38 signaling demonstrating that HO-1 regulates apoptosis as well as p38 signaling during injury [103]. Increased intracellular ratios of NAD(+)/NADH were observed in renal tissues of mice treated with an NQO-1 activator, β-lapachone, relative to cisplatin alone. Furthermore, alleviation of inflammatory cytokines and markers of renal damage were also noted in the β-lapachone cotreatment compared to the cisplatin alone group. Interestingly, in a xenograft model, treatment with β-lapachone enhanced the efficacy of cisplatin indicating that intracellular regulation of NAD(+) levels through NQO1 activation might be a favorable target for the protection against cisplatin-induced acute kidney injury [104]. GPx is a selenoprotein and as such selenium supplementation is known to increase the expression and activity of GPx [108]. Studies have shown that a deficiency of selenium decreases the activity of GPx resulting in increased oxidative stress and contributes to cisplatin-induced nephrotoxicity [106]. Conversely, supplementation with selenium partially ameliorated cisplatin-induced nephrotoxicity [106]. Our group has previously shown that cisplatin treatment results in persistent upregulation of kidney injury markers including Neutrophil Gelatinase associated Lipocalin (NGAL) and Kidney Injury Molecule-1 (KIM1), increased steady-state levels of O_2_^•−^, increased renal tubule damage, and upregulation of mitochondrial electron transport chain complex I activity up to one month following cisplatin treatment [16]. Furthermore, we also demonstrated using a mitochondrial-specific, superoxide dismutase (SOD) mimetic, GC4419, that the disruptions to mitochondrial metabolism that lead to increased mitochondrial O_2_^•−^ following cisplatin administration may be crucial to both AKI and CKD progression [16].

Recent studies reveal increased Nrf2 levels in several cancers whereby the cytoprotective role of Nrf2 promotes cancer cell proliferation thus implying that inhibition of Nrf2 during cancer therapy may be of significance [109]. An inhibitor of Nrf2, Brusatol extracted from Brucea javanica, has been shown to polyubiquitinate Nrf2, decrease cancer cell proliferation, and enhance the efficacy of chemotherapeutic drugs [110]. Studies by Hirayama et al. and Tanaka et al. have shown that kidneys from male and female Nrf2-null mice have impaired capacity to scavenge ROS levels [111,112] making them susceptible to cisplatin nephrotoxicity, renal apoptosis, and necrosis [112,113]. Furthermore, treatment with cisplatin increased the mRNA and protein expression of downstream targets of Nrf2 including NQO-1, HO-1, GCLC in WT but not Nrf2-null mice [113]. The same study also showed that treatment with the Nrf2 activator, CDDO-Im [2-cyano-3,12-dioxooleana-1,9-dien-28-oic imidazolide], protected the WT mice from cisplatin-induced toxicity [113]. Studies conducted by Liu et al. in 2009 comparing responses of Nrf2-WT and KO mice in murine models of cisplatin-induced nephrotoxicity showed a decline in renal function, histology, and overall survival in cisplatin treated Nrf2 KO mice [114]. In another study assessing the transcriptional regulation of Nrf2 to mitigate cisplatin-induced nephrotoxicity by Aleksunes et al., extensive nephrotoxicity and increased neutrophil infiltration was observed in plasma and kidney tissues of Nrf2 KO mice following cisplatin treatment [113]. Additionally, increased expression of renal transporters Mrp2 and Mrp4 as well as an increase in the renal mRNA and protein levels of several cytoprotective genes (NQO1, HO-1 and GCLC) was also detected following cisplatin treatment in the Nrf2 WT mice but not in the Nrf2 KO mice [113]. Nrf2 activation has been shown to increase the transcription of anti-inflammatory and antiapoptotic including proinflammatory cytokines, chemokines, and antiapoptotic factors, which can preserve renal function and cellular integrity. A study of intestinal ischemia/reperfusion (IIR) conducted by Tao Meng et al. in 2017 demonstrated that treatment with Nrf2 inhibitors (all-trans retinoic acid or brusatol) increased the expression of inflammatory cytokines as well as proapoptotic proteins Bcl-2, Bax, and caspase-3 [115]. In contrast, t-Butylhydroquinone, an Nrf2 activator, significantly mitigated IIR-induced intestinal injury, decreased the overexpression of inflammatory cytokines, and inhibited the expression of Bax and caspase-3 proteins [115].

## 6. Role of Nrf2 in Autophagy and Cisplatin-Induced Kidney Injury

Nrf2 exerts its influence on autophagy through a range of mechanisms, encompassing the transcriptional regulation of autophagy-promoting genes [116], facilitation of lysosomal biogenesis to ensure efficient autophagosome degradation [117], and orchestration of damaged and dysfunctional mitochondrial removal via mitophagy [118]. The activation of Nrf2 has been shown to upregulate several autophagy-related genes including ULK1, SQSTM1, ATG2B, ATG4D, and ATG5 [116]. Conversely, inhibiting Nrf2 has been associated with reduced expression of autophagy genes [119]. Notably, the disruption of KEAP1-mediated Nrf2 degradation, achieved through the sequestration of Keap1 by p62 into autophagosomes, has also been identified as a means of activating the Nrf2 transcriptional pathway [120]. Moreover, Nrf2 plays a pivotal role in the activation of TFEB/TFE3 [transcription factor EB (TFEB)/transcription factor binding to IGHM Enhancer 3 (TFE3)], the master regulator of lysosomal biogenesis [117]. Recent research by Ong et al. found that Keap1 deficiency induced Nrf2 activation, leading to postdevelopmental lethality characterized by liver lysosome accumulation [117]. This study further elucidated that the loss of Keap1 precipitated aberrant TFEB/TFE3-dependent lysosomal biogenesis [117]. Nrf2 activation has also been implicated in the initiation of mitophagy, coupled with the promotion of mitochondrial biogenesis, and is aligned with the involvement of the mitophagic/autophagic adapter protein sequestosome-1 (SQSTM1/p62), which is a downstream transcriptional target of Nrf2 [118,121].

Autophagy is upregulated in cisplatin-induced acute kidney injury (AKI), leading to a noticeable elevation in p62 levels within renal tissues. Furthermore, the presence of p62 is essential for initiating the autophagic response and facilitating aggresome formation in the context of AKI [122]. In a murine model of cisplatin-induced AKI, a heightened abundance of autophagosomes was observed within the kidney cortex postcisplatin treatment [122]. Notably, the expression of LC3II, a direct marker for mature autophagosomes, exhibited a substantial increase following cisplatin administration, concomitant with elevated levels of Atg5, Atg7, and Becn1—key regulators of autophagosome formation [122]. In a separate study, mice with autophagy deficiency specifically in proximal tubules displayed exacerbated pathology in cisplatin-induced AKI compared to their WT counterparts [123]. This was evidenced by alterations in kidney function, heightened DNA damage levels, intensified p53 activation, increased apoptosis, augmented protein aggregates, and discernible changes in renal morphology [123]. Thus, the multifaceted role of Nrf2 in autophagy encompasses the mitigation of oxidative stress, attenuation of inflammation, and active modulation of the autophagic and mitophagic processes. 

## 7. Redox Based Interventions for Nrf2 Modulation in Cisplatin-Induced Kidney Injury

The complex pathology of cisplatin-induced kidney injury and the persistent nature of cisplatin-induced renal side effects have consistently warranted the need for easily implementable therapeutic interventions and as such numerous redox-based approaches and studies in preclinical models have been implemented to demonstrate the beneficial effects of Nrf2 activation. 

Pharmacological activation of Nrf2 has been investigated preclinically using small molecules activators including sulforaphane [124], dimethyl fumarate [125], and bardoxolone methyl (CDDO-Me) [126] that promote the nuclear translocation of Nrf2 thereby enhancing the expression of the downstream cytoprotective genes thus protecting against renal damage. In a study conducted be Mohammad et al. in 2022, sulforaphane was shown to improve mitochondrial function significantly. Also, it alleviated age-related kidney injury by increasing cortical Nrf2 expression and decreasing the protein expression of the Nrf2 repressor, Keap1 [124]. Another Nrf2 activator, dimethyl fumarate (DMF) is an approved therapeutic for multiple sclerosis [127] and has been shown to ameliorate cisplatin-induced renal tubular injury via increasing the expression of NQO1 and the suppression of inflammatory cytokines including TNF-α and IL-6 thereby reducing the consequent tubulointerstitial fibrosis [125]. The study showed that Wistar rats treated with 7500 ppm (parts per million) DMF in their diet for 5 weeks demonstrated an inhibition of cisplatin-induced outer medullary necrosis and degeneration as well as inhibited interstitial mononuclear cell infiltration [125] suggesting that treatment with an Nrf2 activator mitigates cisplatin-induced renal injury via an antioxidant-based mechanism that involves NQO1. CDDO-Me, a semisynthetic triterpenoid and a potent activator of the Nrf2 pathway, has also been shown to upregulate the antioxidant response and suppress proinflammatory signaling to reduce oxidative stress levels and inflammation in an in vitro model of cisplatin-induced cellular senescence in human proximal tubular cells [126]. Furthermore, it has also been shown to increase the expression of other redox-based, downstream effectors of Nrf2, GCLC, and NQO1 [128].

Supplementary antioxidants including N-acetylcysteine (NAC) [129,130] and alpha-lipoic acid [131] are also being investigated to reduce cisplatin-induced oxidative stress by Nrf2 activation. In the 2019 study by Güntürk et al., rats treated with 250 mg/kg NAC along with 10 mg/kg cisplatin showed a significant increase in the levels of myeloperoxidase-1 (MPO1) and high mobility group box-1 (HMGB-1) as well as ameliorated structural and functional changes in renal tissues [130]. Additionally, preclinical studies with α-lipoic acid (LA) suggested protection against cisplatin cytotoxicity via activation of the Nrf2/HO-1 pathway [131]. LA was also shown to induce the nuclear translocation of Nrf2 as well as include the expression of SOD1 protecting mice against oxidative stress [131]. 

Several dietary phytochemicals and natural compounds have been investigated as modulators of Nrf2 for prevention against cisplatin-induced injury including quercetin [132], resveratrol [133], curcumin [134], and catechins. Quercetin, a bioflavonoid shown to activate Nrf2 and HO-1 expression [135], has anti-inflammatory, antioxidant, and anticancer activities [136]. Quercetin mitigates cisplatin-induced nephrotoxicity in Fischer rats with breast adenocarcinoma without compromising the antitumor activity [132]. This study showed that treatment with quercetin markedly decreased levels of urinary KIM-1 and Gamma-glutamyl transpeptidase (GGT) suggesting decreased tubular damage [132]. Curcumin, another dietary antioxidant with nephroprotective properties, has been shown not to protect against cisplatin-induced kidney damage by ameliorating the levels of TNF-α, IL-6, KIM-1, NGAL, and Bax/Bcl-2 ratio in renal tissues [134]. 

## 8. Limitations and off Target Effects of Nrf2 in Kidney Injury

With “targeting of Nrf2 pathway” emerging as an attractive therapeutic approach, several aspects related to safety and efficacy remain unclear with regards to the clinical potential of Nrf2. While the activation of Nrf2 leads to cytoprotection and a significant reduction in cisplatin-induced kidney injury [113,114], it can also confer resistance in cancer cells, potentially compromising the effectiveness of chemotherapy [137,138]. Thus, designing interventions that target Nrf2 need to be selective in protecting normal cells whilst proving efficacious in cancer cell killing. Due to the comprehensive nature of the Nrf2 pathway and its downstream effectors, modulation may present with off-target effects which could lead to inadvertent consequences or impede normal cellular physiology. Tumor heterogeneity is another aspect that needs to be considered in Nrf2 activation. Studies have shown that constitutive activation of Nrf2 promotes cancer development as well as contributes to cancer cell resistance [139]. In the clinical setting, the exacerbated expression of Nrf2 has been linked with poor prognosis [139,140,141]. Nrf2 signaling pathway can be constitutively activated in cancers in several ways including mutations in oncogenic Myc, KRas (Kirsten rat sarcoma) and BRaf (v-raf murine sarcoma viral oncogene homolog B1) via mitogen-activated protein kinases (MAPKs) [142], mutations in Keap1, Cul3, or Nrf2 leading to disruptions in the Keap1/Nrf2 axis [140], and DNA methylation of Keap1 reducing Keap1 expression [143]. Persistent activation of the Nrf2 pathway can thus contribute to uncontrolled cancer cell proliferation, enhanced chemoresistance, and angiogenesis leading to metastasis. It is important to note that, while the sustained activation of Nrf2 may interfere with the cytotoxic effects of cisplatin on cancer cells, the transient activation of Nrf2 may not be substantial enough to protect kidneys from cisplatin’s side effects.

Additionally, the activation/suppression of Nrf2 may have global systemic effects thus influencing several organ systems including the liver [144], lung [145], and immune system. Therefore, expected systemic consequences need to be considered without impacting overall treatment outcomes. With a plethora of promising preclinical evidence preceding the translation of Nrf2-targeted therapies and activators to clinical settings, critical factors such as patient age, tumor heterogeneity, bioavailability, drug delivery, and pharmacokinetics need to be considered to ensure the efficacy and safety of Nrf2 modulation approaches in patients.

## 9. Nrf2-Targeted Cancer Clinical Trials and Kidney Outcomes

Alterations in Nrf2 expression and its downstream targets has been observed in many cancers and as such in recent years, there has been a growing interest in targeting the Nrf2 signaling pathway for cancer therapy. Whilst the activation of Nrf2 has been focused on as a therapeutic approach, it is important to note that consistent upregulation of Nrf2 in the kidney has been associated with the development of kidney complications including diabetic nephropathy [146]. The current clinical trial landscape based on Nrf2 modulation primarily focuses on assessing overall survival, safety, and efficacy of the treatment, and so the adverse events associated with kidney injury are often not the primary endpoint. Nevertheless, there are several clinical trials that have investigated the combination of Nrf2 modulation during cisplatin treatment. A plethora of molecules often described as inducers and/or activators of Nrf2 are available in the literature but only a few have been worked on in the clinic including DMF, Oltipraz, SFN, and CDDO-Me which are all based on the interactions of Nrf2 with Keap1 through cysteine151 which is a redox sensor [147,148].

CDDO-Me, a potent Nrf2 activator that acts through the electrophilic modification of Keap1-Cys-151, was initially tested in a phase 1 clinical trial for advanced solid tumors and lymphoid malignancies (NCT00529438, NCT00508807). Administered orally once daily for 21 days of a 28-day cycle, 10 patients out of 44 (five melanoma, two renal cell carcinomas, and one each thyroid, colorectal, and lymphoma) experienced disease stabilization for 4 to 10 months while two patients underwent partial/complete responses. The authors confirmed Nrf2 activation through qRT-PCR analysis of the Nrf2 target gene, NQO1, and found significant increases on day 2 and day 22. On retrospective analysis of the study, it was found that at 900 mg/d there was a statistically significant increase in eGFR (33.9 ± 4.2%; *p* < 0.0001) on day 21 compared with baseline, suggesting that CDDO-Me might reduce rates of CKD [149].

Sulforaphane (SFN), an isothiocyanate found in cruciferous vegetables, has also been reported as a potent Nrf2 activator through direct modification of Keap1 or by inhibiting histone deacetylase (HDAC). Several clinical trials investigating SFN have been initiated in breast (NCT03934905), prostate (NCT01950143), and melanoma (NCT01568996). While the trials showed mixed results in terms of cancer tissue biomarker changes and efficacy of the treatment, no significant changes in biomarkers of kidney function or adverse kidney events were reported, suggesting that adjuvant treatment with SFN does not impair kidney function [150,151,152]. 

Metformin, a biguanide traditionally used to control blood glucose, has been increasingly investigated in terms of cancer therapy and has complex mechanisms associated with the Nrf2 signaling pathway. Metformin has been found to activate Nrf2/Keap1 signaling in neurons, the liver, and skeletal muscle in an AMPK-dependent manner [153,154] however, it has also been found to inhibit Nrf2 and reverse chemoresistance in various cancer cell lines [155,156]. The use of metformin is absolutely contraindicated in patients with severe CKD (eGFR < 30 mL/min/1.73 m^2^) due to the risk of the life-threatening complication, lactic acidosis [157].

The importance of Nrf2 signaling in cancer development, progression, and treatment has led to a wide array of clinical trials in which modulation of Nrf2 has been evaluated. While these trials have produced mixed results in terms of cancer therapy, they often highlight the importance of Nrf2 signaling in terms of adverse events related to radiochemotherapy in normal tissues, including the renal system. Careful consideration must be given when targeting Nrf2 in cancer therapy, but there is wide evidence that its modulation has an effect on renal outcomes during and following therapy. 

## 10. Conclusions

Given the differential biological effects of Nrf2 in normal vs. cancer cells, specific modulations of the Nrf2 pathway should be carefully considered when treating with chemotherapeutic agents like cisplatin. The activation of Nrf2 in normal cells leads to enhanced expression of NQO-1, HO-1, and other antioxidant and cytoprotective genes that can aid in scavenging ROS and reduce oxidative stress and levels of inflammatory proteins. Both pharmacological and genetic interventions have been used in the past to modulate Nrf2 signaling in normal cells. Although the significance of Nrf2 activation lies in ameliorating adverse events following cisplatin treatment in the kidney, there is some preclinical evidence that Nrf2 can induce resistance to cisplatin. Thus, the question of when, how, and where Nrf2 inducers/activators can be employed during cancer therapy becomes of importance to prevent chemoresistance in “at risk” patient populations. Overall, the Nrf2 signaling system is a redox-based sensor that plays a crucial role in responses to cancer therapy-induced stress and should be intently targeted and modulated to ensure better clinical outcomes. 

## Data Availability

No new data were created or analyzed in this study. Data sharing is not applicable to this article.
